# High FGF‐21 level in a cohort of 22 patients with Dravet Syndrome—Possible relationship with the disease outcomes

**DOI:** 10.1002/epi4.12534

**Published:** 2021-09-29

**Authors:** Anna Ka‐Yee Kwong, Virginia Chun‐Nei Wong, Sheila Suet‐Na Wong, Vanessa Loi‐Yan Chu, Saskia Koene, Jan Smeitink, Cheuk‐Wing Fung

**Affiliations:** ^1^ Department of Paediatrics and Adolescent Medicine Li Ka Shing Faculty of Medicine The University of Hong Kong Hong Kong SAR China; ^2^ Department of Paediatrics and Adolescent Medicine Hong Kong Children’s Hospital Hong Kong SAR China; ^3^ Radboud Centre for Mitochondrial Medicine Department of Paediatrics Radboud Institute for Molecular Life Sciences Radboud University Nijmegen Medical Centre Nijmegen The Netherlands

**Keywords:** dravet syndrome, epileptic encephalopathy, FGF‐21, fibroblast growth factor 21, mitochondrial oxidative phosphorylation, valproate

## Abstract

**Objective:**

Dravet syndrome (DS) is a severe and intractable form of epilepsy with prolonged seizures which may evolve to other seizure types and associated with mild‐to‐severe intellectual disabilities. Fibroblast growth factor 21 (FGF‐21) is a stress hormone mediating metabolic and oxidative stress and circulating level of FGF‐21 had been shown to increase in some patients with impairment of oxidative phosphorylation in muscles. In DS, FGF‐21 is of interest for further study as mitochondrial oxidative stress was identified previously in patients.

**Methods:**

Plasma FGF‐21 levels were compared between 22 DS patients and 22 normal controls, and their clinical characteristics of DS patients at the time of plasma sampling were studied retrospectively. Besides, the relationships of FGF‐21 level with intellectual development, seizure frequency, valproate treatment, and types of *SCN1A* mutations were analyzed. Logarithmic transformation of FGF‐21 levels was performed before comparison and statistical analysis.

**Results:**

Mean of log_10_ FGF‐21 level was significantly higher in DS patients when comparing with normal controls (*P* = .0042). Mean of log_10_ FGF‐21 level was significantly higher in DS patients with normal‐to‐mild ID versus mild‐to‐severe ID (*P* = .0193) and with valproate treatment versus without valproate treatment (*P* = .015). No significant difference was shown in FGF‐21 level in DS patients with missense versus truncating *SCN1A* variants, and no correlation could be demonstrated between seizure frequency and FGF‐21 level.

**Significance:**

Significantly higher level of plasma FGF‐21 was identified in DS patients. The high FGF‐21 levels were shown to be associated with developmental outcome and valproate treatment. These results support further investigation on the relationship of FGF‐21 with the clinical outcomes of DS and other related mechanism which is important for possible therapeutic development for this epileptic encephalopathy.

**Key Points**

1. We measured plasma FGF‐21 level in patients with Dravet syndrome (DS) and studied their clinical characteristics.
2. Plasma FGF‐21 level was significantly higher in patients with DS when comparing with normal controls.
3. FGF‐21 levels were significantly higher in DS patients with normal‐to‐mild intellectual disability and valproate treatment.
4. These results support further investigation on the relationship of FGF‐21 with the clinical outcomes of DS and possible therapeutic development.

## 1 INTRODUCTION

Dravet syndrome (DS) is a severe form of epileptic encephalopathy with prolonged febrile or afebrile seizures which may evolve to other seizure types.^1^, ^2^ Overall, DS is poorly responsive to antiseizure drugs (ASDs). DS patients have seizure onset in the first year and developmental delay from the second year of life. Most patients have mild‐to‐severe intellectual disabilities. The majority (70%‐80%) was found to have pathogenic variants in the *SCN1A* gene encoding the α1 pore‐forming subunit of the voltage‐gated sodium channel.^3^ The degree of developmental delay in DS can be associated with the types of *SCN1A* variants as reported in our previous study of 18 DS cases^4^ and other developmental modifiers such as defective mitochondrial oxidative phosphorylation found in other studies.^5^, ^6^

Fibroblast growth factors (FGFs) are a family of polypeptide growth factors which mediate various biological processes including embryonic development, angiogenesis, tissue repair, homeostasis, and metabolism regulation.^7^, ^8^ In vertebrates, 23 family members (FGF1‐23) have been identified^9^ and they are structurally related signaling molecules participating in different pathophysiological processes by paracrine and endocrine secretion. FGF‐21 is a circulating cytokine acting as a metabolic regulator for glucose and lipid metabolism.^10^ It is suggested to be a stress hormone‐mediating responder to metabolic and oxidative stress.^10^, ^11^, ^12^ Elevation of serum FGF‐21 was found in patients with impairment of oxidative phosphorylation in muscle, and thus, FGF‐21 had been proposed as a biomarker for muscle‐manifesting mitochondrial diseases.^13^ Kim et al.^11^ speculated that FGF‐21 may be an adaptive regulator to counteract muscle stress caused by mitochondrial dysfunction based on the demonstration of positive effect of FGF‐21 on mitochondrial function and oxidative capacity.^14^ The brain is particularly vulnerable to mitochondrial oxidative stress and previous in vitro and in vivo studies had been demonstrated that FGF‐21 might possess a neuroprotective role in central nervous system.^15^, ^16^

As elevation of circulating FGF‐21 had been documented in patients with muscle‐manifesting mitochondrial diseases, we tried to investigate the FGF‐21 level in other severe neurological diseases and found that it was also elevated in DS. In this study, we have retrospectively measured the FGF‐21 concentration of stored plasma in a small cohort of DS patients and normal controls. FGF‐21 levels in DS patients versus normal controls and the FGF‐21 level in DS patients with different clinical features including intellectual development, seizure frequency, ASDs, and the types of *SCN1A* mutations were then compared. High FGF‐21 levels in DS patients and their correlation with clinical outcome suggest further studies on the neuroprotective role of FGF‐21 as a possible biomarker or therapeutic agent in DS.

## 2 METHODS AND MATERIALS

Twenty‐two patients diagnosed to have DS were enrolled from the Neurology Clinic of Queen Mary Hospital and Duchess of Kent Children’s Hospital. The normal controls consisted of 22 age‐matched healthy children.

The DS patients were identified based on International League Against Epilepsy (ILAE) classification (2017)^1^ and core DS phenotypes defined by Dravet.^2^ Intellectual disability (ID) was assessed according to their scores of developmental/intelligence quotients as mild ID (quotient = 50‐70), moderate ID (quotient = 25‐50), or severe ID (quotient < 25).

The genetic information of 15 DS patients in our cohort has been reported in our previous study.^4^ For the remaining 7 DS patients, all exons covering the coding regions, as well as the splice junctions of *SCN1A,* were amplified by polymerase chain reaction (PCR) and sequenced. Variant analysis was performed by alignment with the reference genomic sequences (GeneBank accession no.: NG_011906). Variants were discriminated from single nucleotide polymorphisms (SNP) reported in NCBI SNP and Ensembl SNP database. Missense variants were predicted to be deleterious to protein function by homology‐based tool PolyPhen‐2 (http://genetics.bwh.harvard.edu/pph2/) and SIFT (http://sift.bii.a‐star.edu.sg/). Splice site variants in intron regions were analyzed by another online software tool, the Automated Splice Site Analyses (https://splice.uwo.ca/). MLPA was performed in one DS patient using SALSA MLPA probemix (P137‐B2, MRC‐Holland, Amsterdam, The Netherlands).

Plasma samples were previously collected from DS patients and normal controls. Samples were stored at −80°C until analysis. Plasma FGF‐21 concentrations were measured in duplicate by enzyme‐linked immunosorbent assay (ELISA) for FGF‐21 (BioVendor, Brno, Czech Republic) according to the manufacturer’s instructions. The FGF‐21 concentrations were calculated from the standard curve constructed from standards provided by the kit.

Plasma FGF‐21 levels were compared between 22 DS patients and 22 normal controls and the clinical characteristics of DS patients at the time of plasma sampling were studied retrospectively. The FGF‐21 levels were compared between DS patients with mild‐to‐normal versus moderate‐to‐severe intellectual disabilities, with versus without valproate treatment, with missense versus truncated *SCN1A* variants. Logarithmic transformation of FGF‐21 levels was performed before comparison of the means of two groups by unpaired *t*‐test. The correlation seizure frequency with the transformed FGF‐21 levels was statistically tested by Pearson correlation analysis. Besides, seizure frequency was compared between the DS patients with and without valproate treatment by unpaired *t*‐test. Chi‐squared test was employed to illustrate the association of valproate treatment (with and without valproate) versus the degree of ID (normal‐to‐mild ID versus moderate‐to‐severe ID). Since this is an exploratory study, two‐tailed *P*‐values <.05 were considered significant. All statistical analysis was performed using SPSS software version 23 (IBM Corp., USA).

This study was approved by the Institutional Review Board of the Hong Kong West Cluster and the University of Hong Kong (IRB Ref. No.: UW 11‐190 and UW 13‐443).

## 3 RESULTS

The cohort of 22 DS patients were all confirmed to have *SCN1A* mutations. Fifteen DS patients in our cohort have been reported to have pathogenic *SCN1A* variants in our previous study,^4^ 6 DS patients were also identified with missense, splice site *SCN1A* variants, and the remaining one was found to have deletion of exon 14‐16 in *SCN1A* gene (Table 1). The normal controls consisted of 22 healthy children, age ranging from 2 months to 15 years (Table S1), were matched with the age of the DS patients ranging from 2 years 8 months to 23 years (Table 1). The plasma FGF‐21 concentrations and the clinical characteristics including intellectual disability, seizure frequency at the time of plasma sampling of the DS patients were also shown in Table 1.

**TABLE 1:** Clinical features of 22 patients with Dravet syndrome (DS)

Case	Age at plasma sampling	FGF‐21 (pg/mL)	Intellectual disability	Seizure frequency per month	Type of seizures	SCN1A variants	Valproate treatment (+/−)	Other concomitant ASDs
6	22 y	80.6	Moderate	2.5	Generalized tonic seizures	Frameshift c.2971_2972delinsG, p.(Leu991Valfs*2)	−	Clobazam, Topiramate, Phenytoin
24	14 y	544.2	Moderate	1	Generalized tonic clonic seizures	Splice site IVS21 + 1G > A	+	Clobazam
40	9 y	0 (lower than detectable level)	Moderate	<1	Multiple seizure types (Generalized tonic clonic, typical absence, generalized onset myoclonic, focal aware seizures)	Missense c.2378C > T, p.(Thr793Met)	−	Clonazepam, Keppra
46	9 y	330.6	Mild	<1	Multiple seizure types (Generalized tonic clonic, typical absence, generalized onset myoclonic, focal aware seizures)	Missense c.311C > T, p.(Ala104Val)	+	Only Valproate has been used
53	7 y	977.4	Normal	2	Generalized tonic clonic seizures	Nonsense c.1348C > T, p.(Gln450*)	+	Clobazam
65	8 y	40.6	Mild	6.5	Generalized tonic clonic seizures	Missense c.4834G > A, p.(Val1612Ile)	−	Clobazam, Levetiracetam, Carbamazepine
71	15 y	8.6	Moderate	4	Generalized tonic clonic seizures	Nonsense c.569G > A, p.(Trp190*)	+	Lamotrigine, Clobazam, Lorazepam
74	11 y	151.0	Moderate	3.5	Generalized tonic clonic seizures	Missense c.2214G > A, p.(Trp738*)	+	Topiramate
75	13 mo	783.3	Mild	9	Multiple seizures type (Focal aware motor, focal impaired awareness motor, generalized tonic clonic seizures)	Missense c.1177C > T, p.(Arg393Cys)	+	Topiramate
76	9 mo	24.5	Normal	2.5	Generalized tonic clonic seizures	Missense c.1264G > A, p.(Val422Met)	−	Phenobarbitone, phenytoin
77	3 y	1712.1	Mild	1.5	Generalized tonic clonic seizures	Frameshift c.4229delA, p.(Asn1410Metfs*2)	+	Clobazam, Levetiracetam
85	23 y	72.4	Moderate	11	Generalized tonic clonic seizures	Nonsense c.1053T > A, p.(Cys351*)	−	Clobazam, Topiramate, Levetiracetam
89	2 y 8 mo	34.3	Mild	7	Generalized tonic clonic seizures	Splice site IVS3 + 3A > C	−	Clobazam, Oxcarbazepine
94	4 y	6378.6	Mild	2	Recurrent febrile status epilepticus with generalized tonic clinic seizures, focal to bilateral tonic clonic seizure, absence seizures.	Missense c.3641T > G, p.(Ile1214Arg)	+	Clobazam
100	13 mo	270.1	Normal	1.5	Generalized tonic clonic seizures	Frameshift c.4558delC, p.(Gln1520Lysfs*19)	+	Clobazam
101	11 y	289.3	Moderate to severe	16	Generalized tonic clonic seizures	Splice site IVS24‐1G > T	+	Clobazam
102	4 y	2921.2	NA	0	Generalized tonic clonic seizures, focal aware motor seizure	Missense c.380A > T; p.(His127Leu)	+	Clobazam, Stiripentol
103	19 mo	4409.9	Normal	1	Focal aware motor seizure, focal impaired awareness motor seizures	Missense c.965G > T, p.(Arg322Ile)	+	Zonisamide
104	6 y	774.3	Normal	3	Generalized tonic clonic seizures	Missense c.4988T > G, p.(Leu1663Trp)	+	Levetiracetam
105	4 y	5180.4	Mild	0	Generalized tonic clonic seizures	Splice site IVS12 + 2T > A	−	Clobazam
106	3 y	111.8	Moderate	1	Generalized tonic clonic seizures	Nonsense c.3611G > A; p.(Trp1204*)	+	Clobazam, Topiramate
107	2 y 10 mo	769.5	Normal	3.5	Generalized tonic clonic seizures	Deletion from exon 14 to exon 16	+	Clobazam

y = years; mo = months; GGT = gamma‐glutamyl transferase (values expressed as IU/L); ALT = alanine aminotransferase (values expressed as IU/L); NA: clinical information is not available; ASDs: antiseizure drugs. We confirm that we have read the Journal’s position on issues involved in ethical publication and affirm that this report is consistent with those guidelines.

**FIGURE 1:**
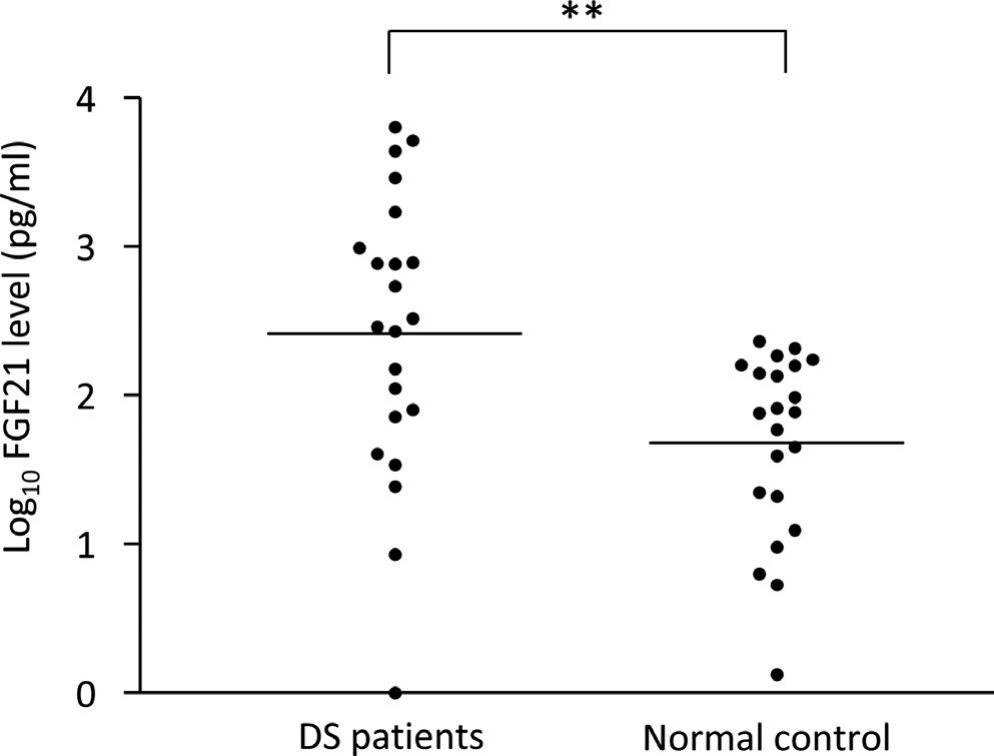
Comparison of plasma FGF‐21 concentrations (pg/mL) in patients with Dravet syndrome (DS) and normal control. Mean of log_10_ FGF‐21 level were significantly higher in DS patients comparing with normal controls by unpaired *t*‐test with 2‐tailed *P*‐values <.01**. Horizontal lines indicated mean of log_10_ FGF‐21

Plasma FGF‐21 concentration ranged from 1.3 to 231.7 pg/mL in normal controls and ranged from 0 to 6378.6 pg/mL in DS patients. When comparing between DS patients and normal controls, mean of log_10_ FGF‐21 level was significantly higher in DS patients (Mean ± SEM: 2.415 ± 0.2043; n = 22) than normal controls (Mean ± SEM: 1.681 ± 0.1309; n = 22) by unpaired *t*‐test (two‐tailed *P* value = .0042) (Figure 1).

**FIGURE 2:**
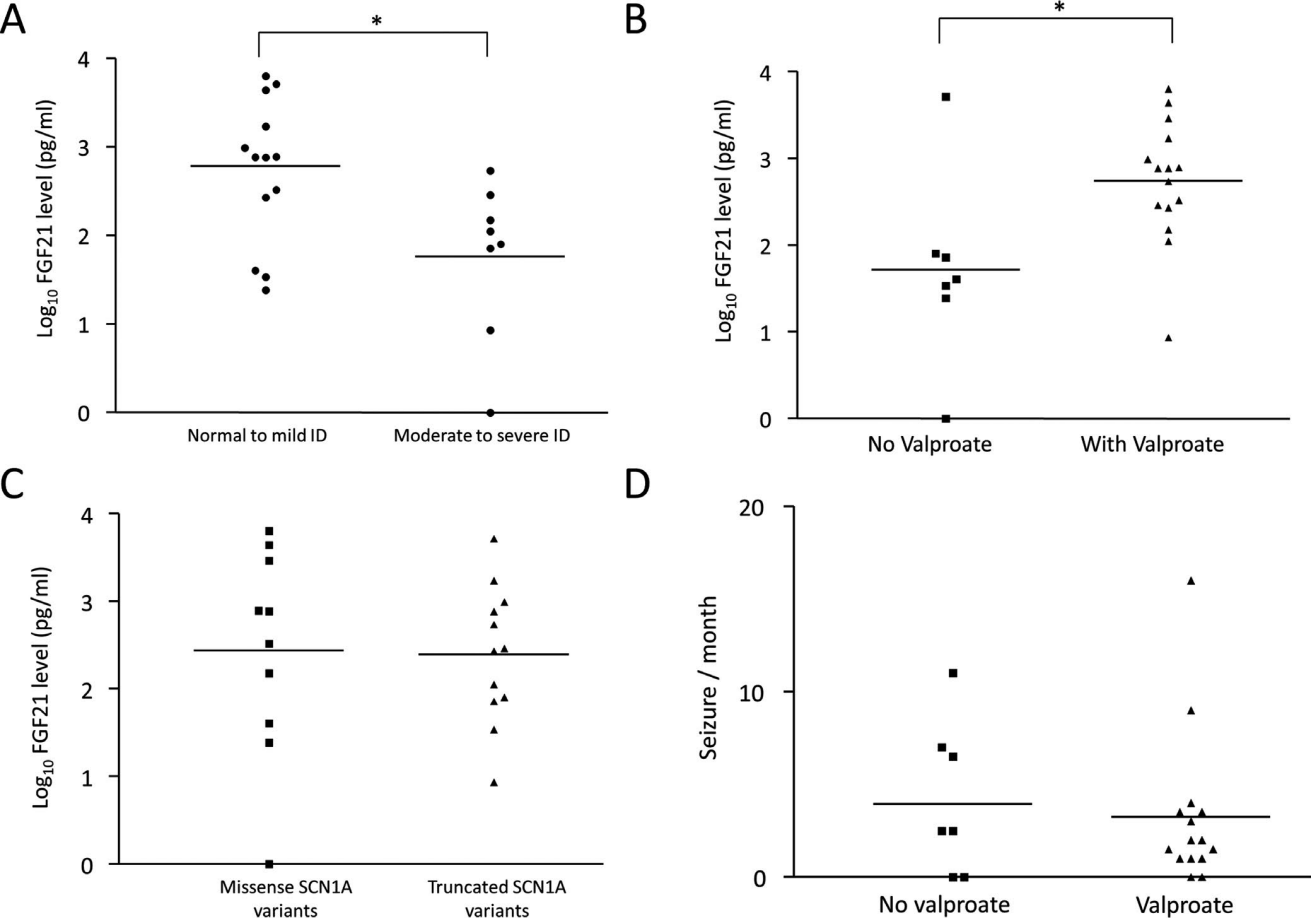
Comparison of plasma FGF‐21 concentrations (pg/mL) and seizure frequency in patients with Dravet syndrome (DS) (A) Log_10_ FGF‐21 level in DS patients with mild‐to‐normal versus moderate‐to‐severe intellectual disabilities (ID); (B) Log_10_ FGF‐21 in DS patients with versus without valproate treatment; (C) Log_10_ FGF‐21 level in DS patients with missense versus truncating *SCN1A* variants; (D) Seizure per month in DS patients with versus without valproate treatment. Mean of log_10_ FGF‐21 level of the two groups were compared using unpaired *t*‐test and 2‐tailed *P*‐values <.05* were considered significant. Horizontal lines indicated mean of log_10_ FGF‐21

When comparing among 22 DS patients, mean of log_10_ FGF‐21 level was significantly higher in DS patients with normal‐to‐mild ID (Mean ± SEM: 2.734 ± 0.2261; n = 13) comparing with those with moderate to severe ID (Mean ± SEM: 1.766 ± 0.3138; n = 8) by unpaired *t*‐test (two‐tailed *P* value = .0193) (Figure 2A). In addition, the mean of log_10_ FGF‐21 level was significantly higher in DS patients with valproate treatment (Mean ± SEM: 2.741 ± 0.1842; n = 15) comparing with those without valproate treatment (Mean ± SEM: 1.716 ± 0.4125; n = 7) by unpaired *t*‐test (two‐tailed *P* value = .015) (Figure 2B). Moreover, treatment with valproate did not result in major liver function derangement. For *SCN1A* variants, mean of log_10_ FGF‐21 level of DS patients with missense variants (Mean ± SEM: 2.439 ± 0.3741; n = 10) has no significant difference from those with truncated variants (Mean ± SEM: 2.395 ± 0.2249; n = 12) by unpaired *t*‐test (two‐tailed *P* value = .916) (Figure 2C). There is no significant association between valproate treatment (with versus without valproate treatment) and the developmental outcome (normal‐to‐mild versus moderate‐to‐severe ID) of the DS patients by chi‐square analysis.

**FIGURE 3:**
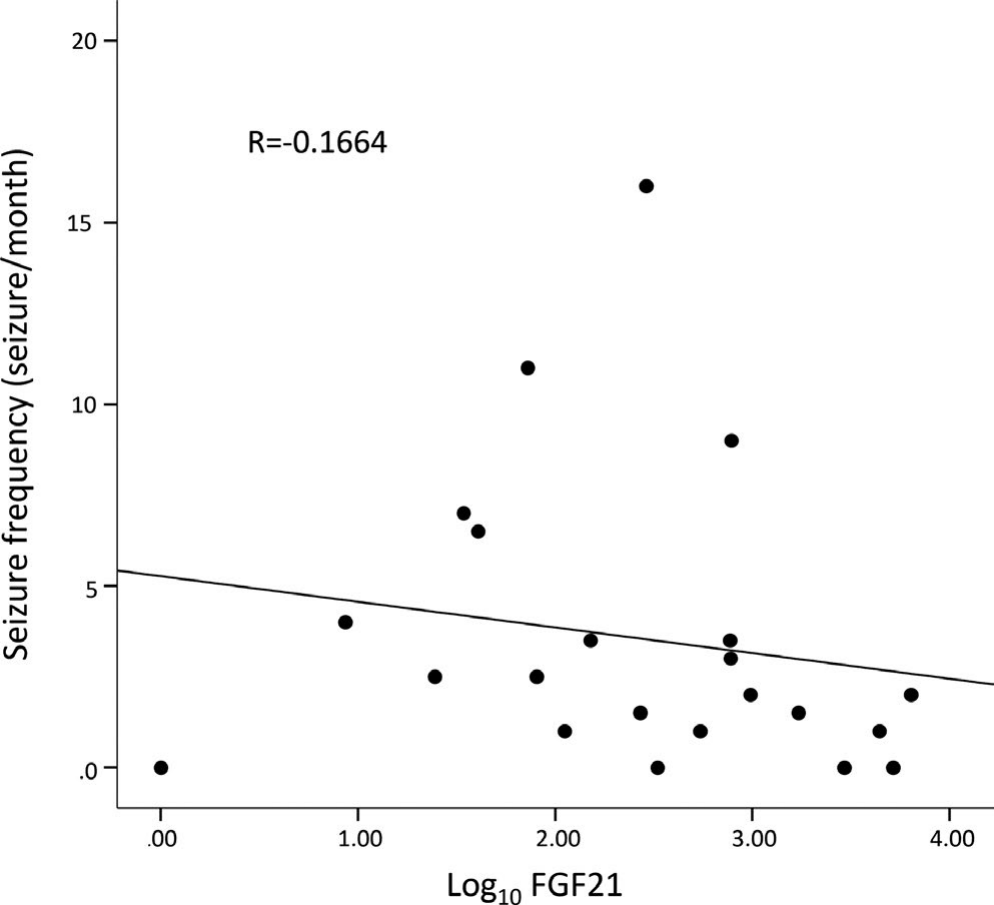
Correlation of seizure frequency and plasma FGF‐21 concentrations in patients with Dravet syndrome (DS)

When considering the seizure frequencies of DS patients at the time of plasma sampling, there was no correlation between number of seizures per month and log_10_ FGF‐21 level (Pearson correlation coefficient = −0.1664, *P* = .459) (Figure 3). There is no significant difference in seizure frequency of DS patients with valproate treatment (Mean ± SEM: 3.267 ± 1.073; n = 15) and without valproate treatment (Mean ± SEM: 4.214 ± 1.546; n = 7) by unpaired *t*‐test (two‐tailed *P* value = .6223) (Figure 2D).

## 4 DISCUSSION

### 4.1 Relationship of high FGF‐21 with oxidative stress

We found a significantly higher plasma FGF‐21 level in the DS patients when comparing with normal controls and some levels in DS patients were very high which is comparable to those in patients with oxidative phosphorylation disorders.^13^ Recently, mitochondrial bioenergetics and gluconeogenesis were proven to be impaired in a zebrafish model of DS.^17^ Previous studies had also shown mitochondrial oxidative phosphorylation dysfunction in^5^, ^6^ 2 DS patients who fulfilled the adult diagnostic criteria of mitochondrial disease.^5^, ^18^ Another study showed a low cellular complex III amount, severe decline in complex III activity and significant reduction of mitochondrial ATP content in skin fibroblasts of 4 DS patients.^6^ The study showed no correlation of total mtDNA, mitochondrial haplogroups and *POLG* variants with the defective mitochondrial respiratory chain.^6^ They proposed that cells under oxidative stress in DS increase free radical production which worsens clinical phenotypes in addition to the effect of the Nav 1.1 defect. Mitochondrial oxidative stress of DS patients, therefore, may be a possible reason for the stimulation of circulating FGF‐21. Further investigation should be considered to understand whether oxidative stress and mitochondrial dysfunction are involved in DS patients. Skin fibroblast cells from the DS patients or mice with *SCN1A* knockout allele can be used as in vitro and vivo models to study the mitochondrial functions including the complex activities, ATP production rate, membrane potential, and reactive oxygen species (ROS) production.

### 4.2 Association of high FGF‐21 with clinical outcome

In the present study, clinical outcomes including seizure frequency and degree of intellectual disability were analyzed retrospectively in DS patients. The FGF‐21 level was significantly higher in the groups of patients with milder ID. Although there was no correlation between seizure frequency and FGF‐21 level by statistical analysis, the patients with higher FGF‐21 level tended to have lower seizure frequencies in our data. It is possible that FGF‐21 acts as an adaptive regulator produced in the DS patients with compensatory response. For those DS patients with even higher FGF‐21 and milder phenotypes, these may be due to the counteracting effect of FGF‐21 on the disease stress to improve the clinical outcome. However, this is only a hypothesis and a more comprehensive study with a larger sampling size should be used to investigate the association of FGF‐21 level with clinical outcomes in DS or other epilepsy syndromes.

FGF‐21 was considered to be an important regulator of mitochondrial oxidative stress and had therapeutic potential to attenuate cell damage and death, endoplasmic reticulum stress, and inflammation.^12^ Previous study suggested that FGF‐21 increase is a compensatory response elicited by mitochondrial respiratory chain deficiency to enhance mitochondrial function in skeletal muscle.^19^ In central nervous system, FGF‐21 was also suggested to have neuroprotective function. In human dopaminergic neuronal cells, FGF‐21 was shown to increase mitochondrial respiratory capacity, biogenesis and functions.^20^ Study of obese, insulin resistant rat model illustrated the effect of FGF‐21 on prevention of cognitive decline which is associated with its abilities to improve hippocampal synaptic plasticity, dendritic spine density, brain mitochondrial function, and attenuate brain cell apoptosis.^16^ If there are more evidence to prove the association of FGF‐21 level with clinical outcomes, further studies could be done to investigate the mechanism of FGF‐21 induction in DS patients and correlation with milder ID. It remains to be discovered whether upregulation of circulating FGF‐21 may be a compensatory mechanism to protect the neuronal cells from severe damages under metabolic or oxidative stress in DS patients.

### 4.3 Effect of valproate treatment to FGF‐21 level

We also found that FGF‐21 levels were significantly higher in DS patients who received valproate treatment. In vitro studies had illustrated that valproate upregulated FGF‐21 expression in primary neurons or cortical glial cells.^15^, ^21^ Another study on adult patients with depressed bipolar disorder received valproate treatment (500‐1000 mg daily) for 12 weeks showed a significant increase in FGF‐21 level from 167.7 to 207.1 pg/mL.^22^ However, this increase in circulating FGF‐21 was not as high as those found in our DS cohort. Nevertheless, valproate may not be the sole factor to induce FGF‐21 but may possibly act synergistically with other factors such as mitochondrial oxidative stress in DS to trigger the release of FGF‐21. In another recent study, valproate was suggested to have a neuroprotective effect through epigenetic mechanisms by modulation of histone deacetylases (HDACs) influencing different gene expression^23^ and *FGF21* is one of the genes suggested to be upregulated by valproate through HDACs.^21^

### 4.4 Limitations

The retrospective nature is the limitation of the present study. One should set up a standard sampling protocol for FGF‐21 measurement at different time courses to understand the variation of FGF‐21 level in different clinical conditions such as seizure frequency, intellectual development, or clinical responses to ASDs. The time of plasma sampling is important to be fixed because of the diurnal rhythm of circulating FGF21 as suggested in a previous study.^24^

As FGF‐21 may be elevated in obesity,^25^ nutrition and body mass index could be potential confounding factors that may affect the metabolic status and hence the FGF‐21 levels. Such factor would have to be further investigated in the future studies to prove the direct relationship between DS and FGF‐21.

### 4.5 Clinical relevance or future directions

FGF‐21 has the potential to be developed as a biomarker to monitor the severity and treatment response in DS if a comprehensive study with a larger sampling size can be further carried out. In addition, the neuroprotective mechanisms of FGF‐21 and the relationship with mitochondrial dysfunction can be further investigated for a possible therapeutic target for DS or other epilepsy syndromes.

## 5 CONCLUSION

In conclusion, we identified significantly higher plasma FGF‐21 levels in DS patients when compared with normal controls. Besides, the high FGF‐21 levels were shown to be associated with better developmental outcomes and valproate treatment. Further study on the relationship of FGF‐21 with the clinical outcomes and other related mechanisms will be important for possible therapeutic development for DS. Use of FGF‐21 as a potential predictor of neurodevelopmental outcome may be considered in our clinical practice.

## CONFLICT OF INTEREST

Anna Ka‐Yee Kwong, Virginia Chun‐Nei Wong, Wong Sheila Suet‐Na, Vanessa Loi‐Yan Chu, Saskia Koene, and Cheuk‐Wing Fung declare that they have no conflict of interest. Jan Smeitink is the CEO of Khondrion, a pharmaceutical company developing compounds to potentially treat mitochondrial disease.

## Supporting information

Table S1
