# Supplementary material for: High FGF‐21 level in a cohort of 22 patients with Dravet Syndrome—Possible relationship with the disease outcomes
Source: Epilepsia Open. 2021 Sep 29;6(4):685–93. doi: 10.1002/epi4.12534 (PMC8633467; doi:10.1002/epi4.12534)
Supplement: Supplementary file 1 — Table S1 [file EPI4-6-685-s001.docx]

**Supplementary table 1**

**Age and plasma FGF-21 concentration of 22 normal controls**

| **Normal control** | **Age at plasma sampling** | **FGF-21 (pg/ml)** |
| --- | --- | --- |
| C1 | 11y | 77.2 |
| C2 | 3y | 97.1 |
| C3 | 15y | 135.4 |
| C4 | 10y | 82.2 |
| C5 | 2m | 160.9 |
| C6 | 6y | 159.7 |
| C7 | 2y | 185.0 |
| C8 | 7y | 45.2 |
| C9 | 12y | 175.0 |
| C10 | 4y | 231.7 |
| C11 | 3y | 12.5 |
| C12 | 11y | 21.0 |
| C13 | 2y | 5.4 |
| C14 | 15y | 208.0 |
| C15 | 12y | 1.3 |
| C16 | 14y | 141.2 |
| C17 | 2y | 9.6 |
| C18 | 2y | 76.4 |
| C19 | 8y | 59.1 |
| C20 | 5y | 22.3 |
| C21 | 11y | 39.2 |
| C22 | 14y | 6.3 |
